# Transcriptome Analysis of the Global Response of *Pseudomonas fragi* NMC25 to Modified Atmosphere Packaging Stress

**DOI:** 10.3389/fmicb.2018.01277

**Published:** 2018-06-11

**Authors:** Guangyu Wang, Fang Ma, Xiaojing Chen, Yanqing Han, Huhu Wang, Xinglian Xu, Guanghong Zhou

**Affiliations:** ^1^Key Laboratory of Meat Processing and Quality Control, Nanjing Agricultural University, Nanjing, China; ^2^College of Veterinary Medicine, Nanjing Agricultural University, Nanjing, China; ^3^Jiangsu Collaborative Innovation Center of Meat Production and Processing, Quality and Safety Control, College of Food Science and Technology, Nanjing Agricultural University, Nanjing, China; ^4^Jiangsu Province Physical and Chemical Testing Center, Nanjing, China

**Keywords:** *Pseudomonas fragi*, modified atmosphere packaging, spoilage, transcriptome, cell metabolism

## Abstract

*Pseudomonas fragi* is usually isolated from chilled meats in relation to their spoilage, while many studies have shown that the application of modified atmosphere packaging (MAP) inhibits the spoilage potential of *P. fragi*. The effects of MAP on *P. fragi* NMC25 metabolism were determined in the present study by exposing this organism to different air conditions and comparing the resulting transcriptome profiles. We found 559 differentially expressed genes by RNA-seq, and the results revealed that MAP decreases the expression of genes involved in the electron transport chain (*nuoAB*), resulting in an inhibition of aerobic respiration. Meanwhile, MAP also induced the downregulation of genes responsible for ATP-binding cassette transporters, flagellar and type I fimbrial proteins, and DNA replication and repair, which may further influence nutrient uptake, motility, and growth. In addition, NMC25 cells modified their pathways for energy production, amino acid synthesis, membrane lipid composition, and other metabolic patterns to adapt to MAP. These data show that *P. fragi* NMC25 survives under MAP but reduces part of its metabolism related to its spoilage ability.

## Introduction

*Pseudomonas* spp. constitute the main spoilage microorganism of meat stored in aerobic refrigerated conditions (Nychas et al., 2008; Mohareb et al., 2015; Rouger et al., 2017). These species are responsible for meat physical damage, off-odor production, and slime formation. *Pseudomonas fragi* is currently recognized as the most abundant member of the *Pseudomonas* spp. on fresh meat, which is a particularly suitable substrate for *P. fragi* growth (Ercolini et al., 2010). In addition to taking advantage of the nutritional value of the meat components, *P. fragi* can easily proliferate during the chill chain applied to meat production. Moreover, *P. fragi* is suggested to promote the growth and survival of several foodborne pathogens, such as *Staphylococcus aureus* and *Listeria monocytogenes* (Casaburi et al., 2011).

Spoilage is often associated with bacterial enzyme functions or metabolic activities. Researchers are attempting to correlate these bacterial metabolic activities with spoilage appearance. Previous studies showed that spoilage bacteria produce malodorous volatile compounds through their ability to metabolize amino acids and other substances (Samelis, 2006; Casaburi et al., 2015). Jääskeläinen et al. (2015) found that the production of acetoin and diacetyl, which cause the buttery off-odor of meat, is related to the utilization of various carbon sources.

However, environmental changes have a significant impact on the biological functions of microorganisms. The development of high-throughput sequencing helps people further understand the molecular response of bacteria to different environments. With an emphasis on these issues, many investigations have focused on the effects of environmental factors such as nutrient composition, growth phases, temperature, pH, and osmotic stress (Hazen et al., 2015; Andreevskaya et al., 2016; Han et al., 2017; Jia et al., 2017; Koh et al., 2017). Environmental changes may also alter the metabolism of spoilage bacteria and thus influence the spoilage process. Modified atmosphere packaging (MAP) is an effective method for extending the shelf lives of various fresh meats. Previous studies in our research group have demonstrated that MAP can affect bacterial surface properties and synthesis of extracellular polymeric substances (Wang et al., 2017a,b). Fully understanding the mechanisms of these phenotypic changes could lead to decreasing the role of *P. fragi* in meat spoilage. However, these inhibition mechanisms are still unclear. Modulation of gene expression has a central role in cellular responses to environmental stress, with extensive regulation occurring at the transcriptional level. Identifying differentially expressed genes (DEGs) that underlie these phenotypic changes is therefore crucial.

*Pseudomonas fragi* NMC25 was isolated from spoiled meat and showed strong spoilage potential. The aim of this study is to provide a global view of gene expression in *P. fragi* NMC25 in response to MAP conditions, leading to understanding the cellular strategies that are employed under MAP and to proposing a potential mechanism for the inhibition of spoilage activity under MAP.

## Materials and Methods

### Bacterial Strains and Culture Conditions

*Pseudomonas fragi* NMC25, a strain with strong spoilage potential, was previously isolated and identified from spoiled chilled chicken (Wang et al., 2017a,c). The isolate was subcultured twice from frozen (-80°C) glycerol stocks in tryptone soy broth (TSB) for 24 h at 28°C. Stationary-phase cells were harvested by centrifugation at 10,000 ×*g* for 10 min at 4°C, washed twice, and resuspended in 0.9% NaCl solution.

The MAP used in this study was performed by a SMART 500 (ULMA Packaging, Barrio Garibai, Spain) under a modified atmosphere containing 70% N_2_ and 30% CO_2_ using Lid 1050 MAP films (Cryovac, Sealed Air Co., Ltd, Shanghai, China). The film has an oxygen permeability of 6 cm^3^ m^-2^.day^-1^.atm^-1^ at 0% relative humidity/4°C and a water vapor permeability of 0.1 g m^-2^.day^-1^ at 100% relative humidity/4°C. The detailed MAP information can be found in **Supplementary Figure S1**. Air was used as a control atmosphere. All samples were stored at 15°C.

### Meat Contamination and Sample Collection

Meat samples (10 g) were sliced from chilled chicken breasts after they had been sterilized via irradiation at a dose of 6 KGy (Wang et al., 2017c). The surfaces of meat pieces were inoculated with a stationary-phase culture of *P. fragi* NMC25 at a concentration of 3 log CFU/g. All the samples were packaged and stored as described above. Late-exponential phase cultures (1.5 days for air samples and 3.5 days for MAP samples) were harvested (Wang et al., 2017b) and immediately used for RNA extraction. For the collection of strains grown on meat, the dispersed biomass on each meat sample was gently harvested using a polypropylene inoculating loop and then suspended in 0.9% NaCl solution. The experiments were performed using three independent replicates.

At the sampling date, we analyzed the gas composition of the headspace in MAP using an OXYBABY gas analyzer. The gas composition was 0.53% O_2_, 27.3% CO_2_, and 72.15% N_2_, suggesting that the film was a high oxygen barrier film.

### RNA Extraction, Library Construction, and Sequencing

Total RNA was isolated using the Trizol Reagent (Thermo Fisher Scientific, United States). The quantity and integrity were determined using NanoDrop ND-2000 spectrophotometer (Thermo Fisher Scientific, United States) and Agilent 2100 Bioanalyzer (Agilent Technologies, Santa Clara, CA, United States). The total RNA was depleted of rRNA using a Ribo-Zero rRNA Removal Kit (Illumina, San Diego, CA, United States) and then fragmented into short fragments. First strand cDNA was synthesized using random oligonucleotides and SuperScript III, followed by the synthesis of second strand cDNA using DNA polymerase I and RNase H. Exonuclease and polymerase were used to blunt and adenylate the 3’ ends of the DNA fragments, and Illumina PE adapter oligonucleotides were ligated to prepare for hybridization. To select cDNA fragments around 300 bp in length, the library fragments were purified using the AMPure XP system (Beckman Coulter, Beverly, CA, United States). DNA fragments with ligated adaptor molecules on both ends were selectively enriched using Illumina PCR Primer Cocktail in a 15-cycle PCR reaction. The products were purified with the AMPure XP system and quantified using the Agilent 2100 Bioanalyzer. Finally, the cDNA library was then sequenced using the Illumina NextSeq 500 platform (Personal Biotechnology Co., Ltd. Shanghai, China).

### Analysis of the DEGs

The raw reads were filtered to obtain the high-quality clean data by removing adaptor sequences and low-quality reads with Q-value ≤ 20. The clean reads were then mapped to the *P. fragi* NMC25 complete genome (NCBI reference sequence, NZ_CP021132) using Bowtie2 v.2.2.4^1^. Reads per kilo base per million reads (RPKM) values were calculated and normalized to transform into expression values by using Rockhopper ^2^. Finally, differential expression analysis for RNA-seq data was also performed by Rockhopper. The resulting *p*-values were adjusted for controlling the false discovery rate. The DEGs were sorted based on expression ratios ≥ 2.0-fold and adjusted *p*-values < 0.05. To better understand the biological functions and the metabolic pathways of the identified genes, the DEGs were functionally classified according to the Gene Ontology (GO) and Kyoto Encyclopedia of Genes and Genomes (KEGG) databases. The GO Slim was created by the Perl script map2slim.pl ^3^ available from the GO Consortium. Another Perl script was written to establish associations between DEGs and the pathway information from KEGG database.

The raw transcriptome reads reported in this paper have been deposited in the NCBI Sequence Read Archive with accession number SRP133716.

### Validation of the DEGs Using qRT-PCR

To validate the RNA-seq data, quantitative reverse transcription PCR (qRT-PCR) analyses of selected genes were performed. The total RNA was extracted as described above and reverse transcribed into cDNA. Quantitative PCR was performed using SYBR green (Prime Script RT Master Mix, TAKARA, China) on a 7500 Fast Real-time PCR System (Applied Biosystems, Foster, CA, United States). The primers designed for RT-qPCR in this study are listed in **Supplementary Table S1**. Levels of target gene transcripts were calculated relative to the 16S rRNA using the 2^-ΔΔCt^ method to normalize expression levels. All samples were analyzed in triplicate.

## Results

### General Transcript Features

**FIGURE 1:**
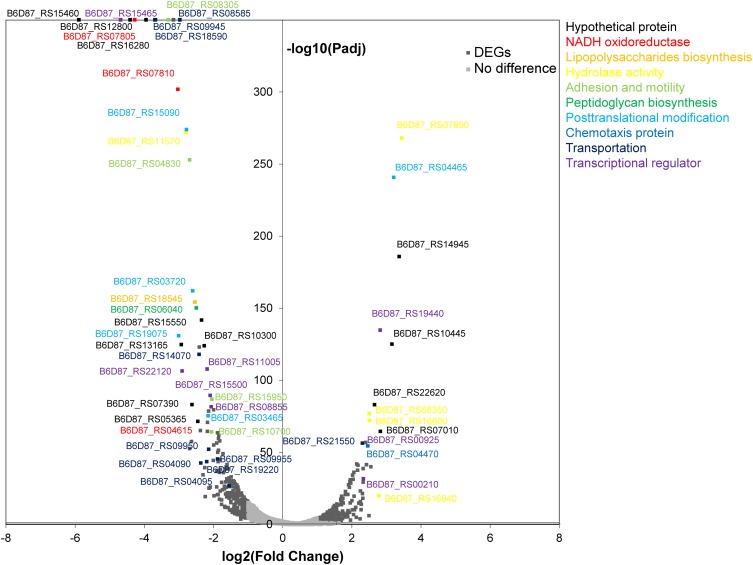
Volcano plot of genes expressed differentially between MAP and air samples. *P*_adj_, adjusted *p*-value. The *P*_adj_ of the DEGs at the top is 0 (all data corresponding to DEGs under these two conditions can be found in **Supplementary Table S2**).

To compare the lifestyle of *P. fragi* NMC25 under air and MAP, we compared the transcriptome profile of cells growing on meat under MAP with that of cells growing under air using RNA-seq. A complete table of the DEGs and their relative fold changes at each time point can be found in **Supplementary Table S2**. The transcriptomic analyses indicate that growth under MAP led to changes in the expression of at least 559 genes, which is almost 12% of the genome. The expression of 294 genes (approximately 53%) decreased under MAP, while that of 265 other genes (approximately 47%) increased. A total of 52 transcriptional regulators were identified in DEGs, indicating a clear response to the environment. Genes with significant alterations were mainly categorized as hypothetical proteins, NADH oxidoreductases, chemotaxis proteins, and transcriptional regulators or related to lipopolysaccharide biosynthesis, hydrolase activity, motility and adhesion, peptidoglycan biosynthesis, post-translational modification, and transport (**Figure 1**). The expression of genes coding for proteins involved in NADH oxidoreductases, motility and adhesion, and transport was strongly repressed under MAP. Moreover, the overall fold change in downregulated genes was larger than that in upregulated genes. The differentially expressed transcripts were subjected to GO analysis and consequently classified into three major functional categories and 58 subcategories (**Supplementary Figure S2**). The transcriptome revealed that biological processes composed the largest proportion of the three functional categories and that biosynthetic processes and cellular nitrogen compound metabolic processes were two most abundant subgroups in the biological process category, expect for genes with unknown functions. The biological functions associated with DEGs were further analyzed using the KEGG database. A total of 218 DEGs were mapped to 19 different KEGG pathways. Three categories contained the majority (55.05%) of the regulated genes with annotations: amino acid metabolism, global and overview maps, and carbohydrate metabolism (**Supplementary Figure S3**).

Although genes implicated in many other cellular functions were affected, many of the DEGs were involved in energy production, amino acid metabolism, transportation, motility and adhesion, and DNA replication and repair.

### Functional Analyses of DEGs Related to Cell Metabolism

**FIGURE 2:**
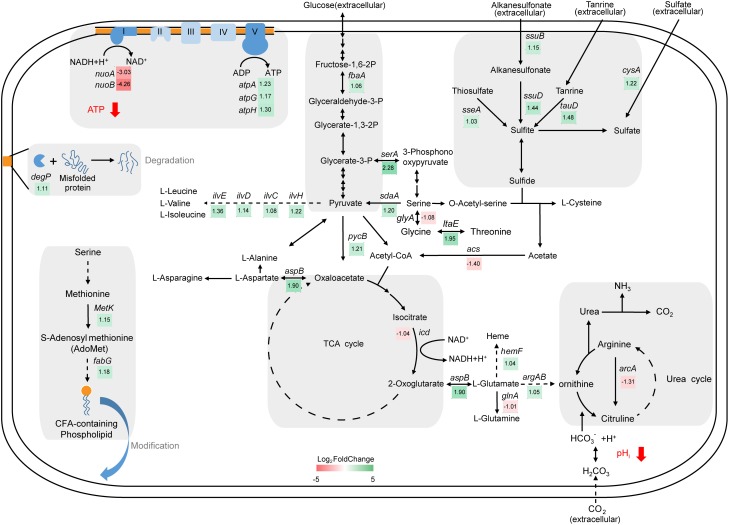
Depiction of biological process changes (mainly in energy production and amino acid metabolism) in MAP samples based on the Kyoto Encyclopedia of Genes and Genomes (KEGG) database. Red indicates a lower expression level in MAP samples than in controls, and green indicates a higher expression level in MAP samples.

Growth under MAP induced changes in the expression of genes related to glycolysis, the tricarboxylic acid (TCA) cycle, oxidative phosphorylation, and ATP-binding cassette (ABC) transporters (**Figure 2** and **Table 1**). The upregulation of aldolase (*fbaA*) in the glycolytic pathway may lead to an accumulation of pyruvate. Pyruvate can be used to construct several amino acids. The expression of a series of genes involved in amino acid biosynthesis, including that of serine, valine, leucine, isoleucine, cysteine, asparagine, alanine, and ornithine, was also higher in the MAP samples. The accumulation of serine is involved in upregulating the synthesis of S-adenosylmethionine (AdoMet). In addition, the expression of *sseA, ssuB, ssuD, tauD*, and *cysA* proteins, which are involved in sulfur metabolism, was higher in MAP samples. In constrict, isocitrate dehydrogenase (*icd*), which mediates the TCA cycle, was expressed less in MAP samples. The NADH generated by the TCA cycle or other biological processes can be fed into the oxidative phosphorylation pathway. The expression of complex I subunits (*nuoA* and *nuoB*) in the oxidative phosphorylation pathway was significantly decreased in MAP samples, with log2 fold changes of 3.03 and 4.26 from their control values, respectively. Moreover, several of the components of the ABC transporters, which mediate the recruitment of molecules from outside cells, were downregulated, and some were upregulated. Specifically, most of the ABC transporter proteins that were downregulated were permeases or substrate-binding proteins, while the upregulated proteins were ATP-binding proteins.

**Table 1:** Differentially expressed genes related to the transport of amino acids or peptides.

Gene ID	Symbol	Log_2_ fold change	FDR	Product or description
Down				
B6D87_RS09945	*gltK*	-3.68326	0	Glutamate/aspartate import permease protein
B6D87_RS09950	*aapQ*	-2.13465	7.47E-53	General L-amino acid transport system permease protein
B6D87_RS09955	*glnH*	-1.87801	4.27E-46	Amino acid ABC transporter substrate-binding protein
B6D87_RS12000	*ddpB*	-1.02046	2.31E-12	Peptide ABC transporter permease
B6D87_RS08585	*AzlC*	-2.96592	0	Branched-chain amino acid ABC transporter permease
B6D87_RS04090	*dppF*	-2.36453	2.54E-43	ABC transporter ATP-binding protein
B6D87_RS04095	*dppD*	-1.54057	6.24E-27	Dipeptide ABC transporter ATP-binding protein
B6D87_RS08405	*HisJ*	-1.1635	1.86E-15	Histidine ABC transporter periplasmic binding protein
Up				
B6D87_RS01815	*gltI*	1.21723	7.65E-06	Glutamate/aspartate periplasmic binding protein
B6D87_RS09370	*yhdZ*	1.12749	9.13E-06	Amino acid ABC transporter ATP-binding protein
B6D87_RS20640	*braF*	1.28011	1.27E-04	ABC transporter ATP-binding protein
B6D87_RS21550	*gltL*	2.30834	4.20E-57	Glutamate/aspartate import ATP-binding protein
B6D87_RS21925	*argT*	1.3356	1.19E-04	Lysine/arginine/ornithine-binding periplasmic protein

**FIGURE 3:**
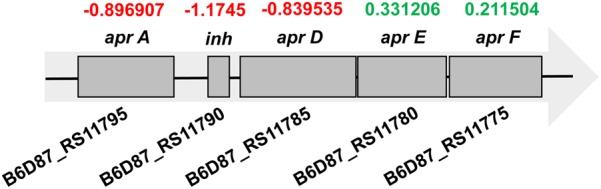
Gene expression pattern of the alkaline protease operon in MAP samples. Numbers above each gene are the log_2_ fold change values relative to transcription in controls. Red and green colors represent downregulation and upregulation, respectively.

Alkaline proteases (APRs) in *Pseudomonas* spp. are secreted by a type I secretion pathway. **Figure 3** shows that the gene expression patterns of APR operons were not significantly changed. The expression of *aprA, aprD*, and the inhibitor *inh* was downregulated slightly in MAP samples, while the expression of *aprE* and *aprF* was slightly increased.

Motility and adhesion play important roles in bacterial biofilm formation. In this study, the expression of genes related to motility and adhesion was altered after MAP exposure. **Table 2** shows that the expression of several genes associated with flagella (*flgB, fliF, fliJ, fliO, fliP*, and *motA*) was significantly decreased in MAP samples. The fimA gene, which encodes type I fimbrial protein, was also downregulated under MAP.

**Table 2:** Differentially expressed genes related to motility and adhesion.

Gene ID	Symbol	Log_2_ fold change	FDR	Product or description
B6D87_RS10585	*fliF*	-1.23704	1.00E-21	Flagellar M-ring protein
B6D87_RS14560	*motA*	-1.05585	3.27E-10	Flagellar motor protein
B6D87_RS14670	*fliP*	-1.05828	4.65E-07	Flagellar biosynthetic protein
B6D87_RS14675	*fliO*	-1.29389	6.46E-23	Flagellar biosynthetic protein
B6D87_RS14720	*fliJ*	-1.07009	6.81E-16	Flagellar biosynthesis chaperone
B6D87_RS14800	*flgK*	-1.18845	1.57E-22	Flagellar hook-associated protein
B6D87_RS19000	*fimA*	-1.4233	6.39E-10	Type 1 fimbrial protein
B6D87_RS10700	*flgB*	-2.05352	6.68E-65	Flagellar basal-body rod protein

Four DEGs that are involved in DNA replication and repair were identified in the transcriptomes of species under both packaging conditions (**Table 3**). The results confirmed the lower expression levels of exodeoxyribonuclease I (*sbcB*), DNA polymerase III (*dnaQ, dnaX*), and ribonuclease HI (*rnhA*) in MAP samples than controls.

**Table 3:** Differentially expressed genes related to DNA replication and repair.

Gene ID	Symbol	Log_2_ fold change	FDR	Product or description
B6D87_RS16480	*sbcB*	-1.20632	1.88E-04	Exodeoxyribonuclease I
B6D87_RS15665	*dnaQ*	-1.34792	1.26E-15	DNA polymerase III subunit epsilon
B6D87_RS08245	*dnaX*	-1.8529	2.77E-60	DNA polymerase III subunit gamma/tau
B6D87_RS15670	*rnhA*	-2.24793	3.70E-40	Ribonuclease HI

### Validation of DEGs

**FIGURE 4:**
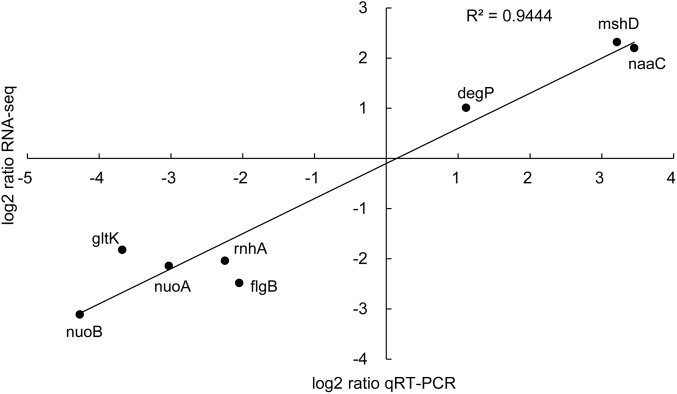
Correlation of gene expression patterns of RNA-seq and qRT-PCR for nine selected genes.

To assess the reliability of RNA-seq data, we performed qRT-PCRs on nine selected genes. **Figure 4** shows that the qRT-PCR data correlate well with the RNA-seq data (*R*^2^ = 0.9444).

## Discussion

Previous studies have demonstrated that MAP can inhibit bacterial growth and prolong the shelf life of chilled meat (Al-Nehlawi et al., 2013; Meredith et al., 2014; Remenant et al., 2015; Zhang et al., 2015). This inhibition has recently been associated with bacterial phenotypic changes under MAP. As phenotypes are related to gene expression patterns, understanding the functions of the genes that are differentially expressed in this bacterium is crucial. The transcriptome profiles of *P. fragi* NMC25 were therefore examined in a food system in this study to determine the changes in gene expression under two different packaging conditions. Furthermore, we performed functional analyses to clarify the possible mechanisms underlying the phenotypic changes from a global perspective.

*Pseudomonas* spp. are generally obligate aerobes. The most obvious repression was thus unsurprisingly found in the oxidative phosphorylation pathway. NADH:ubiquinone oxidoreductases are largely affected by MAP: components of complex I (NuoA and NuoB) were strongly downregulated, but the expression of complex V (F_0_F_1_ ATP synthase subunit alpha, gamma, and delta) was slightly induced (**Figure 2**). Complex I is the largest and most complicated enzyme of the electron transport chain (Brandt, 2006). It links the NADH to ubiquinone electron transfer to the transmembrane transport of protons, contributing to the generation of a proton motive force that is essential for ATP synthesis (Gurrath and Friedrich, 2004). The NuoB subunit in complex I contains the Fe/S cluster N2, which functions as the electron donor to quinone, and loss of NuoB may result in a loss of complex I activity (Flemming et al., 2003). The NuoA subunit is physically and most likely functionally tightly associated with the NuoB subunit (Di Bernardo and Yagi, 2001). The downregulation of NADH: ubiquinone oxidoreductases expression in the present study thus leads to a reduction in the transmembrane gradient and ATP synthesis, which suggests that MAP decreases the amount of cellular ATP in NMC25 cells. Generally, ATP synthesis in prokaryotic organisms occurs mainly by glycolysis using substrate-level phosphorylation and by oxidative phosphorylation. Some *Pseudomonas* spp. (e.g., *Pseudomonas aeruginosa* and *Pseudomonas putida*) can also respire under anaerobic conditions by utilizing nitrate or nitrite (Schobert and Jahn, 2010). In the absence of oxygen, four reductases (nitrate-, nitrite-, NO-, and nitrous oxide reductases) allow bacteria growth (Zumft, 1997). However, the reductase genes (*nar, nir, nor*, and *nos*) required in denitrification were not detected in the genome of *P. fragi* NMC25. To maintain energy consumption, the cell therefore increased glycolysis or other reduction processes and the expression of ATP synthase to produce ATP. In addition, although we used a high oxygen barrier film for MAP (30% CO_2_, 70% N_2_), there still exists 0.53% O_2_ at the sampling date. The level of O_2_ that diffuses through the packaging films/or is trapped within the meat or between the meat slices cannot be completely removed from the packaging system (Ahmed et al., 2017). This microaerophilic condition may also lead to the survival of NMC25.

ATP-binding cassette transporters widely exist in microorganisms and play significant roles in nutrient import (Davidson et al., 2008). They often consist of multiple subunits, including transmembrane proteins and ATP-binding domains. The membrane-spanning region provides a pathway for substrates to cross the cell membrane. In this study, the downregulation of permeases and substrate-binding proteins led to less amino acid or peptide uptake. When growing on meat, *P. fragi* NMC25 mainly uses amino acids and short peptides as its carbon and nitrogen sources. As shown in **Table 1**, the growth of cells under MAP led to decreased expression of genes corresponding to the transport of glutamate, aspartate, peptides, dipeptides, and histidine. Genes related to the transport of general L-amino acids, amino acids, and branched-chain amino acids were also expressed at lower levels in MAP samples. As amino acids are key intermediates in bacterial metabolism, the decreased expression of these permeases and substrate-binding proteins was paralleled by the enhanced expression of genes involved in the biosynthesis of several kinds of amino acids and the ATP-binding proteins of ABC transporters.

In the *P. fragi* NMC25 genome, we identified several open reading frames whose sequences are homologous to the APR system genes of *P. aeruginosa* (locus B6D87_RS11775-B6D87_RS11795). The APR operon includes five genes. *aprA* encodes the structural gene of an APR, and *aprI* encodes a protease inhibitor, but secretion requires three other genes: *aprD* encodes the ABC exporter, *aprE* encodes an accessory factor, and *aprF* encodes an outer membrane factor (Fath and Kolter, 1993). None of the genes in the APR system were expressed differentially. However, the absence of secretion gene functions impairs APR secretion and results in the protein remaining in the cell and being partially degraded (Guzzo et al., 1991). In this study, the lack of ATP may affect the ATP-binding site of the AprD protein, resulting in a delay in the delivery of APRs and their subsequent degradation in the cell. Meanwhile, the upregulated gene *degP* encodes a serine protease that is involved in the degradation of abnormal proteins in the periplasm (**Figure 2**; Waller and Sauer, 1996) and degrades transiently denatured and unfolded or misfolded proteins that accumulate in the periplasm following heat shock or other stress conditions (Isaac et al., 2005). Additionally, molecular interactions with CO_2_ and decreased pH were speculated to inactivate the enzyme (Hu et al., 2013). As a consequence of these combined factors, we thus assume that the extracellular protease activity might be affected by MAP.

Bacteria can form biofilms in various environments, and bacterial motility and adhesion are critical for biofilm formation (O’Toole et al., 2000). Flagellar-mediated motility could enable bacteria to initially reach a surface and then allow them to divide to spread along the surface, while type I fimbriae are required for stable cell-to-surface attachment (Pratt and Kolter, 1998). In this study, several flagellar proteins and one type I fimbrial protein exhibited lower expression in MAP samples. These results indicate that the swimming and biofilm formation ability of *P. fragi* NMC25 would be inhibited under MAP conditions, which agree with our previous study (Wang et al., 2017a).

It has been well established that MAP can inhibit the growth of *Pseudomonas* spp. (Parlapani et al., 2015). In a previous publication, we found that the growth rate of *P. fragi* NMC25 was significantly lower in MAP samples than control samples (Wang et al., 2017b). This result was most likely (except for the ATP issue) ascribed to the significantly lower expression of genes coding for the components of DNA polymerase III, ribonuclease H, and exonuclease I, which are essential for DNA replication and repair. A similar effect has been observed in *Escherichia coli* (Itaya and Crouch, 1991). The colony forming ability of cells was severely affected by a mutation inactivating the ribonuclease H and exonuclease I genes. In addition, the decreased expression of DNA polymerase III also implies that MAP represses the DNA replication of NMC25 cells and thus inhibits cell division.

Nitrogen is an inert gas present in the gas composition used for MAP. Previous studies confirmed that N_2_ treatment decreases the viable cell count little or not at all compared to CO_2_ treatment (Debs-Louka et al., 1999). The ultimate effect of MAP may therefore mainly be caused by CO_2_. CO_2_ can diffuse into the phospholipids of cell membranes and then structurally and functionally disorder the cell membrane (Jones and Greenfield, 1982). Moreover, bicarbonate formed from carbonic acid may also change phospholipid head groups and proteins at the surface of the membrane (Garcia-Gonzalez et al., 2007). In response to these stresses, NMC25 cells intensify the production of AdoMet, whose methyl group can be combined with unsaturated fatty acids (UFAs) for the synthesis of cyclopropane fatty acids (CFAs; Grogan and Cronan, 1997). UFAs play a critical role in maintaining the fluidity of bacterial membranes (Yoon et al., 2015). CFAs have an important role in the survival of bacteria, and a high level of CFA increases the resistance of *E. coli* to environmental (e.g., acid) stress (Chang and Cronan, 1999). NMC25 cells may thus adjust their membrane lipid composition to allow their growth under MAP conditions.

ATP plays an important role in controlling the growth rate and cellular processes, including transportation, central carbon metabolism, molecular synthesis, and motility (Yuroff et al., 2003; Zhou et al., 2009). This study found that the inhibition of nutrient transport, cell division, and motility may also be connected with the synergistic effects of ATP changes. These cellular processes were closely related to the bacterial spoilage potential, and the interruption of these processes could result in the inhibition of spoilage by MAP.

## Conclusion

The current study investigates the molecular response of *P. fragi* NMC25 to MAP. The bacterial cells were packaged in MAP, and an analysis was carried out that compared the transcriptome of these cells with that of cells packaged with air. Most DEGs were involved in energy production, amino acid metabolism, transportation, motility and adhesion, and DNA replication and repair. The suppression of complex I in the electron transport chain, which maintains ATP synthesis and the transmembrane gradient, may be the crucial change caused by MAP exposure. Although the current study has suggested a possible regulatory network of *P. fragi* NMC25 in MAP, the general response of aerobic bacteria maybe more complex and is still unclear. Nonetheless, the RNA-seq data revealed new candidate genes that might provide insight into novel mechanisms involved in the spoilage inhibition caused by MAP.

## Author Contributions

All authors have significant contributions to the completion of the manuscript, final approval, and agreement to be accountable for all aspects of the work. GW and HW conceived and designed the work. GW, FM, and XC performed the data acquisition and analyzed the data. GW, FM, YH, and HW contributed to interpretation of data for the work. GW, HW, XX, and GZ drafted and revised the work.

## Conflict of Interest Statement

The authors declare that the research was conducted in the absence of any commercial or financial relationships that could be construed as a potential conflict of interest. The reviewer RT and handling Editor declared their shared affiliation.

## Footnotes

1 http://bowtie-bio.sourceforge.net/index.shtml

2 http://cs.wellesley.edu/~btjaden/Rockhopper

3 http://search.cpan.org/~cmungall/go-perl/scripts/map2slim
