# Supplementary material for: Transcriptome Analysis of the Global Response of Pseudomonas fragi NMC25 to Modified Atmosphere Packaging Stress
Source: Front Microbiol. 2018 Jun 11;9:1277. doi: 10.3389/fmicb.2018.01277 (PMC6004401; doi:10.3389/fmicb.2018.01277)
Supplement: Supplementary file 1 [file Data_Sheet_1.docx]

**Transcriptome Analysis of The Global Response of *Pseudomonas fragi* NMC25 to Modified Atmosphere Packaging Stress**

Guangyu Wang^a^, Fang Ma^b^, Xiaojing Chen^c^, Yanqing Han^d^, Huhu Wang^a,*^,Xinglian Xu^a,*^, Guanghong Zhou ^a^

a Key Laboratory of Meat Processing and Quality Control, Nanjing Agricultural University, Nanjing, China

b College of Veterinary Medicine, Nanjing Agricultural University, Nanjing, China

c Jiangsu Collaborative Innovation Center of Meat Production and Processing, Quality and Safety Control, Nanjing, China

d The Physical and Chemical Testing Center of Jiangsu Province, Nanjing, China

Running Title: Transcriptome analysis of *P. fragi* under MAP

* Correspondence:

Huhu Wang

huuwang@njau.edu.cn

Xinglian Xu

xlxus@njau.edu.cn

**Supplementary Figures**


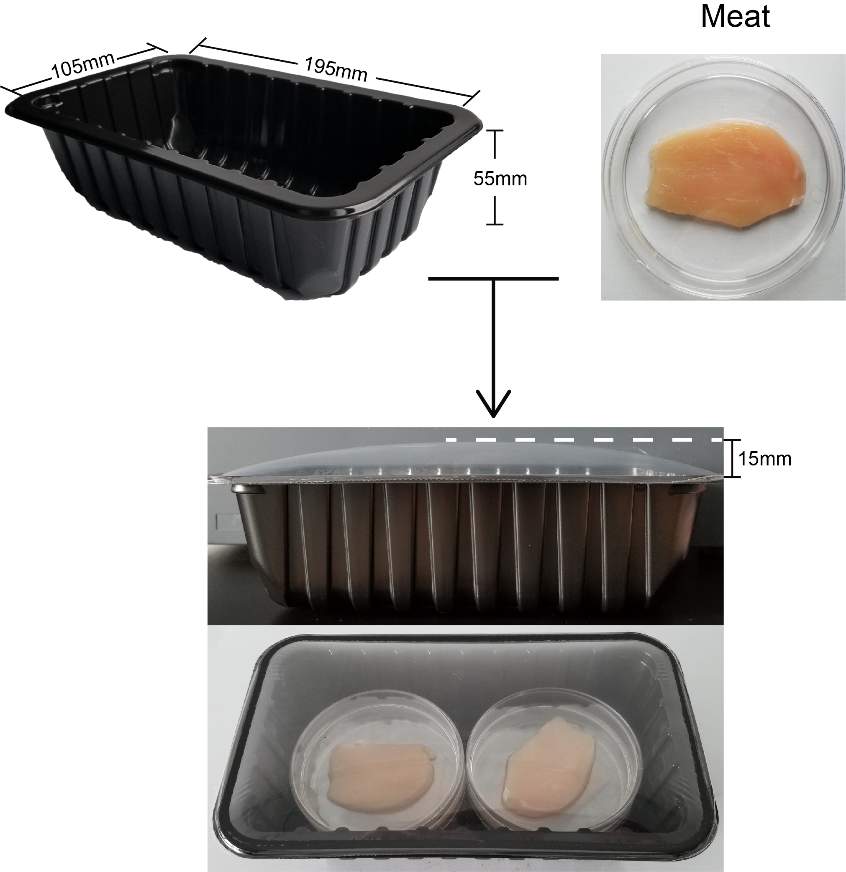


**Supplementary Figure S1.** Packing conditions under MAP.


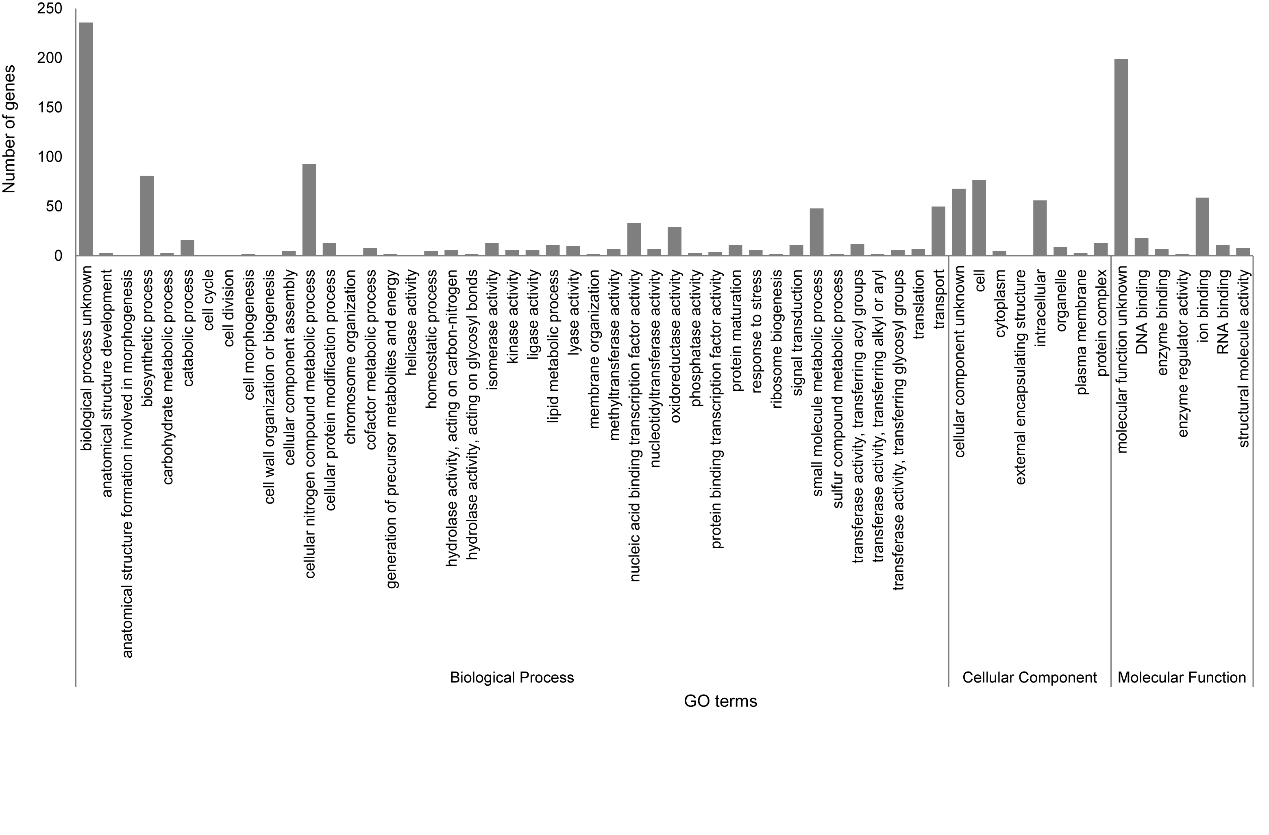
 **Supplementary Figure S2.** Number of DEGs assigned to three GO categories.


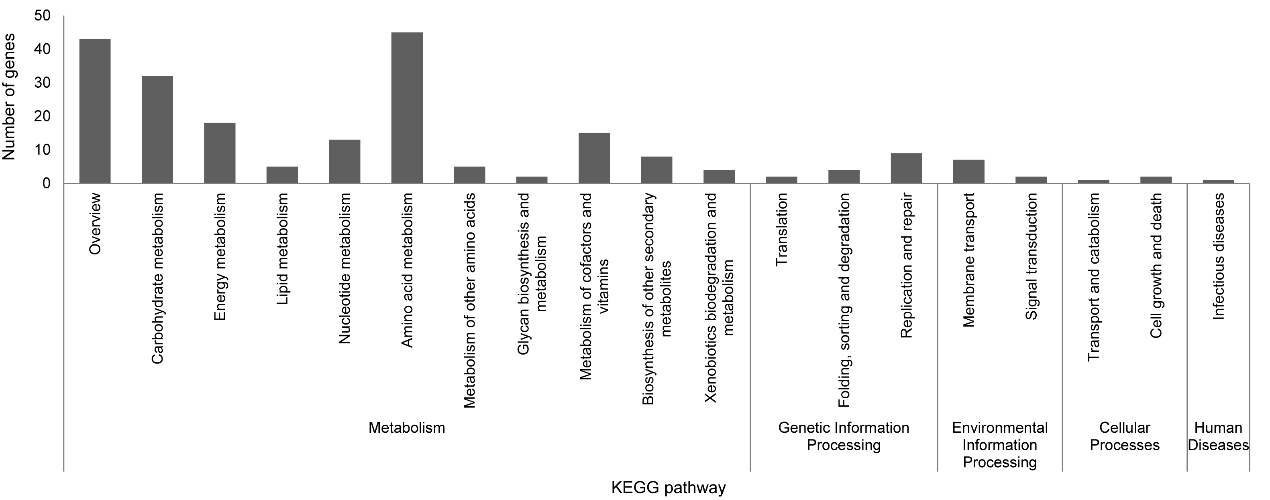


**Supplementary Figure S3.** Number of DEGs assigned to KEGG pathways.
