# Supplementary material for: Transcriptome Analysis of the Global Response of Pseudomonas fragi NMC25 to Modified Atmosphere Packaging Stress
Source: Front Microbiol. 2018 Jun 11;9:1277. doi: 10.3389/fmicb.2018.01277 (PMC6004401; doi:10.3389/fmicb.2018.01277)
Supplement: Supplementary file 2 [file Table_1.DOCX]

**Table S1.** Primers used for qRT-PCR in this study

| Gene ID | Symbol | Gene length | Primer sequence |
| --- | --- | --- | --- |
| B6D87_RS07810 | *nuoA* | 414 | F: 5’-CACCGAGCATGAAGGCACAGAG-3’  R: 5’-CCGAAGCGACAGGACTCATAGC-3’ |
| B6D87_RS07805 | *nuoB* | 675 | F: 5’-CCAGTTGACCGTGCCGTTCAG-3’  R: 5’-AACCGTTGCCGATCCGTTAGAAG-3’ |
| B6D87_RS09945 | *gltK* | 669 | F: 5’-CTCCAGAAAATCAGCATCACC-3’  R: 5’-CAGGGATCATTTCGGCATTG-3’ |
| B6D87_RS21550 | *gltL* | 735 | F: 5’-GTCGAAGTCTTCCAGGCCATTAAGG-3’  R: 5’-GGCGAAGTGCTGGTGGTGATC-3’ |
| B6D87_RS15670 | *rnhA* | 453 | F: 5’-CGAGTCGGTCACCAGCAACAC-3’  R: 5’-CCAACACCACCAACAATCGCATG-3’ |
| B6D87_RS10700 | *flgB* | 351 | F: 5’-GGCTCTCCTGCAATACCGTGTG-3’  R: 5’-TCCGAGGTGCTCTTGGCGAAG-3’ |
| B6D87_RS17245 | *degP* | 1422 | F: 5’-TCTTCTGCTCTTCGGTCAGGTCTG-3’  R: 5’-TGAAGTGATTCGTGACGGCAAGC-3’ |
| B6D87_RS07690 | *naaC* | 729 | F: 5’-GGTTTCTGTTTTGGCGGTTG-3’  R: 5’-GCATCGATAGGGTTAGGTGTG-3’ |
| B6D87_RS04465 | *mshD* | 474 | F: 5’-ACCACCAGGTCCTTGAGGTAAGC-3’  R: 5’-CTGCTGCATGACGCCGAGTAC-3’ |
| B6D87_RS15315 | *16s rRNA* | 1547 | F: 5’-CCTACGGCTACCTTGTTACGACTTC  R: 5’-TCGGAATCGCTAGTAATCGTGAATCAG-3’ |
